# Quantified VMAT plan complexity in relation to measurement‐based quality assurance results

**DOI:** 10.1002/acm2.13048

**Published:** 2020-10-28

**Authors:** Michael Nguyen, Gordon H. Chan

**Affiliations:** ^1^ Department of Medical Physics Juravinski Cancer Centre Hamilton ON Canada

**Keywords:** complexity, patient specific quality assurance, radiotherapy, ROC, treatment verification, VMAT

## Abstract

Volumetric‐modulated arc therapy (VMAT) treatment plans that are highly modulated or complex may result in disagreements between the planned dose distribution and the measured dose distribution. This study investigated established VMAT complexity metrics as a means of predicting phantom‐based measurement results for 93 treatments delivered on a TrueBeam linac, and 91 treatments delivered on two TrueBeam STx linacs. Complexity metrics investigated showed weak correlations to gamma passing rate, with the exception of the Modulation Complexity Score for VMAT, yielding moderate correlations. The Spearman’s rho values for this metric were 0.502 (*P* < 0.001) and 0.528 (*P* < 0.001) for the TrueBeam and TrueBeam STx, respectively. Receiver operating characteristic analysis was also performed. The aperture irregularity on the TrueBeam achieved a 53% true positive rate and a 9% false‐positive rate to correctly identify complex plans. Similarly, the average field width on the TrueBeam STx achieved a 60% true‐positive rate and an 8% false‐positive rate. If incorporated into clinical workflow, these thresholds can identify highly modulated plans and reduce the number of dose verification measurements required.

## 1 INTRODUCTION

Volumetric modulated arc therapy (VMAT) has become a common form of radiation therapy as a result of its ability to deliver highly conformal doses over short delivery times. This is achieved by dynamic multileaf collimator (MLC) movement as well as variable dose rate and gantry speeds.^1^, ^2^ To help identify discrepancies between planned and delivered fluence, patient‐specific quality assurance (PSQA) is often performed, either by measurement in a detector phantom or by independent dose calculations. However, due to the variations in treatment planning systems, delivery systems, and measurement tools, PSQA results between institutions can significantly vary. In light of this, Miften et al.^1^ recently reported on the methodologies of quality assurance practices, recommending tolerance limits for comparison of studies between institutions.

While measurement‐based PSQA is regarded as the most accurate method of assessing delivery accuracy,^1^, ^3^ extensive literature exists in developing pre‐treatment quality assurance (PTQA) tools for this purpose by quantifying plan complexity as indications of dose agreement. These complexity metrics can be used to describe the degree of dose modulation in a treatment. An increased modulation often leads to increased uncertainty in dose because of the limitations of accurately modeling linac components such as MLC tongue and groove which affects the interleaf leakage, and leaf offset between the calculated rounded leaf tip value and the measured one. Generally, higher degrees of modulation suggest more complex treatments, and consequently increased uncertainty in delivery.^3^, ^4^, ^5^, ^6^, ^7^, ^8^, ^9^, ^10^, ^11^, ^12^

Complexity metrics can be used to characterize a treatment plan based on the parameters of the machine used as well as the properties of the treatment plan such as fluence, MLC positions, gantry speed, and dose rate variations. Based on the sources of modulation, complexity metrics can be broadly categorized as fluence map‐based metrics, and aperture‐based metrics.^13^

Fluence map‐based metrics consider the resulting fluence from a given beam or plan. However, these metrics are insensitive to the degeneracy of fluence maps. For example, a fluence map can be the result of a single large beam, or the sum of many small field beams. While the latter may be more mechanically demanding on the linac, a fluence map‐based metric may not always distinguish between these situations.^5^

Aperture‐based metrics generally focus on variations of the MLC positions during delivery.^13^ These metrics can be used to describe the variations in the mechanical and dosimetric machine parameters, noted as deliverability metrics by Chiavassa et al.^3^ Conversely, the MLC alone can be used to describe plan parameters that are likely to compromise accurate dose calculation in the treatment planning system,^3^ or result in disagreements between the treatment planning system and the delivered plan.^13^ This study investigated the use of aperture‐based complexity metrics as PTQA tools with consideration to the recommendations made by Miften et al.^1^ and Chan et al.^14^ at our institution.

## 2 MATERIALS AND METHODS

### 2.A VMAT plans and dose verification

One Varian TrueBeam linac was used to deliver 93 treatments equipped with the Millennium 120 Leaf MLC, while two Varian TrueBeam STx linacs were used to deliver 91 treatments, both equipped with High Definition 120 Leaf MLC. The TrueBeam and TrueBeam STx linacs used different beam models reflecting the differing MLC configurations, and both TrueBeam STx linacs used had been beam‐matched. Table 1 describes the distribution of plans investigated by general treatment site. All treatments considered for this study were randomly selected, delivered using coplanar beams, and clinically approved plans generated in Pinnacle3 (Version 9.10, Philips) using a collapsed cone convolution algorithm. Treatments were delivered at an angular gantry separation of 2°, a maximum dose rate of 600 MU/min, and a nominal energy of 6 MV.

**Table 1:** Distribution of VMAT plans by treatment site.

Treatment Site	TrueBeam	TrueBeam STx
CNS^a^	0	7
GI^b^	8	3
GU^c^	27	22
GYN^d^	8	0
H&N^e^	45	12
Lung	0	37
Other	5	10
Total	93	91

^a^ Central nervous system.

^b^ Gastrointestinal cancer.

^c^ Genitourinary cancer.

^d^ Gynecologic cancer.

^e^ Head and neck.

Dose verification measurements and analysis followed recommendations made by Miften et al.^1^ The measurements were performed using the IBA MatriXX Evolution ion chamber array with a spatial resolution of 7.6 mm to produce 2D planar dose measurements. The detector array was placed in a central cavity of an in‐house polystyrene phantom along the coronal plane. Using the true composite setup, the phantom and detector remained stationary without rotation during measurements. An inclinometer fixed to the linac gantry head was used to correct for the angular dependence of the response of individual ion chamber detectors. Linac output variation was accounted for by delivering 200 MUs on a 10 × 10 cm^2^ field before and after measurements. Isocenter shifts were made as deemed necessary to best represent the clinically relevant regions. The OmniPro ImRT software was used to record measurements and compare the measured dose planes to the Pinnacle3 calculated dose planes via gamma index analysis.

### 2.B Complexity metrics

The degree of complexity of VMAT treatment plans was evaluated using previously established complexity metrics.^3^, ^4^, ^5^, ^6^, ^8^, ^9^, ^10^, ^11^, ^12^ Metrics were selected from those reported to have statistically significant correlations to quality assurance results in previous works, with an emphasis on those describing MLC behavior of the treatment. The following measures were considered:

1. MU Factor, defined as the ratio of the total monitor units to the prescribed dose in cGy.^4^
2. Aperture Irregularity (AI), which describes the aperture shape in relation to a circle.^5^ Irregularly shaped apertures, including off‐central axis fields and small leaf gaps may be more mechanically demanding of the linac to deliver as intended.
3. Modulation complexity score for VMAT (MCSv), which describes the distance traveled by leaf pairs in relation to variations of aperture shape. The MCSv takes a fixed range from 0 to 1.^6^ This metric was adapted from McNiven et al.’s definition,^7^ originally intended for IMRT.
4. Average field width in cm, calculated as the average gap between leaf pairs of a given control point. Average field width per control point is then weighted by the MU to be delivered at each control point.^8^
5. Small aperture score (SAS), defined as the proportion of a plan delivered using small apertures, with a fixed range from 0 to 1. Following the original work, small apertures are defined to be leaf pair gaps less than 2, 5, 10, and 20 mm, resulting in four quantities.^4^

The MU Factor, AI, and SAS are defined to suggest more complex plans with higher values, whereas the MCSv and the Average Field Width indicate more complex plans with lower values.

### 2.C Quality assurance analysis

Gamma index analysis was performed at the 3%/2 mm and 2%/2 mm dose difference and distance to agreement criteria, with a 10% low‐dose threshold and global dose normalization. A tolerance limit indicated by a gamma passing rate (GPR) of 95% is used to distinguish between plans that may be more likely to have dose disagreements between measurement and TPS calculation. Plans with GPRs above the tolerance limit are considered to pass, whereas plans with GPRs below the tolerance limit are considered to fail. The measured dose distribution was captured at the 7.6 mm spacing of the detector, and was the reference distribution for gamma analysis. The dose distribution from Pinnacle3 was calculated at a resolution of 2.5 mm in all dimensions, and cubic spline interpolation was applied to yield a resulting spatial resolution of 0.5 mm in all dimensions to improve the gamma calculation accuracy.^1^ The interpolated planned dose distribution was used as the evaluated dose distribution in gamma analysis.

Internal treatment planning system files containing plan parameters were used to determine complexity metrics for each treatment. An in‐house Python script was used to calculate complexity metrics from planning files as well as to perform statistical analysis. Spearman’s rank correlation coefficient (r_s_) was determined for each pair of GPR and complexity metric to test for the existence of correlations. Strong correlations are indicated as |r_s_| ≥ 0.7, moderate as 0.7 > |r_s_| ≥ 0.5, weak as 0.5 > |r_s_| ≥ 0.3, and no correlation as 0.3 > |r_s_|. Statistical significance of a correlation was taken by a two‐tailed *P* value at *P* < 0.001.

Receiver operating characteristic (ROC) curves were produced to determine if complexity metrics can identify treatment plans with GPRs below the tolerance limit. For each complexity metric, the threshold value used to categorize a given plan to a pass or a fail is varied to determine the true positive and false positive values used in the ROC curves, where a positive result is a failing plan. A true positive is then defined to be a plan with a complexity value less than a given threshold value, and a GPR below the tolerance limit. Similarly, a false‐positive is defined as a plan with a complexity value less than a given threshold value, but a GPR above the tolerance limit.

For example, the MCSv is defined to indicate more complex plans with lower values. With a threshold value of 0.4, a treatment plan with an MCSv of 0.3 and a GPR below the tolerance limit will be considered a true positive occurrence. Similarly, this would be considered a false positive occurrence if the same plan yielded a GPR above the tolerance limit. The MU Factor, AI, and SAS are defined to indicate complex plans at higher values, and require that the complexity value be greater than the threshold value to indicate a plan with a GPR below tolerance as a true positive.

The area under the curve (AUC) for each ROC curve was also determined as an indication of classification performance. The AUC takes values between 0.5 and 1.0, representing chance accuracy and perfect accuracy, respectively. Using the benchmarks presented by Nauta et al.,^9^ a value between 0.5–0.6 is considered poor performance, 0.6–0.7 is fair, 0.8–0.9 is good, >0.9 is excellent, and >0.95 is near perfect performance.

## 3 RESULTS

### 3.A Gamma passing rates

Table 2 describes the distributions of the GPR for the TrueBeam and TrueBeam STx machines, respectively, using both the 3%/2 mm criteria and the 2%/2 mm criteria. For both machines, quality assurance yielded smaller ranges of GPRs using the 3%/2 mm criteria. At this level, all plans investigated had GPRs above the tolerance limit of 95%, indicating all plans would pass quality assurance. As such, the analysis presented in this work focuses on using the 2%/2 mm criteria to include quality assurance results below tolerance. Using this criterion, the TrueBeam delivered 78 passing plans and 15 failing plans, with failures consisting of 7 H&N plans, 5 GU plans, 1 GYN plans, and 2 plans treating other cancer sites. Similarly, the TrueBeam STx delivered 86 passing plans and 5 failing plans, with failures targeting 3 prostate GU plans and 2 lung SBRT plans.

**Table 2:** Descriptive statistics for gamma passing rates.

	TrueBeam (N = 93)		TrueBeam STx (N = 91)	
	3%/2 mm	2%/2 mm	3%/2 mm	2%/2 mm
Number of plans below tolerance	0	15	0	5
Mean GPR	99.4%	97.7%	99.7%	98.9%
Std Dev	0.8%	2.0%	0.7%	1.9%
Min GPR	96.2%	91.2%	95.7%	92.2%
Max GPR	100.0%	100%	100.0%	100.0%

### 3.B Complexity metrics

Table 3 shows the mean values and standard deviations of each complexity metric for plans measured on both the TrueBeam and the TrueBeam STx machines, with distinction for passing and failing plans. Failing plans yielded complexity metric values corresponding to larger disagreements between dose distributions.

**Table 3:** Complexity metrics for all plans analyzed.

Complexity metric	TrueBeam		TrueBeam STx	
	Mean of passes ± SD^a^	Mean of fails ± SD^a^	Mean of passes ± SD^a^	Mean of fails ± SD^a^
MU factor (MU/cGy)	2.4 ± 0.7	3.0 ± 0.8	2.0 ± 0.6	2.7 ± 0.8
AI^b^	7.6 ± 2.9	9.9 ± 2.6	5.4 ± 2.8	6.2 ± 2.2
MCSv	0.40 ± 0.09	0.33 ± 0.04	0.5 ± 0.1	0.39 ± 0.09
Average field width (cm)	3.8 ± 1.3	3.1 ± 0.9	3.3 ± 1.5	2.5 ± 1.1
SAS^c^, 2 mm	0.13 ± 0.05	0.17 ± 0.06	0.11 ± 0.07	0.13 ± 0.06
SAS^c^, 5 mm	0.15 ± 0.06	0.19 ± 0.06	0.13 ± 0.07	0.17 ± 0.08
SAS^c^, 10 mm	0.21 ± 0.08	0.28 ± 0.07	0.2 ± 0.1	0.3 ± 0.1
SAS^c^, 20 mm	0.3 ± 0.1	0.43 ± 0.08	0.4 ± 0.2	0.5 ± 0.2

^a^ Standard deviation.

^b^ Aperture irregularity.

^c^ Small aperture score.

**Fig. 1:**
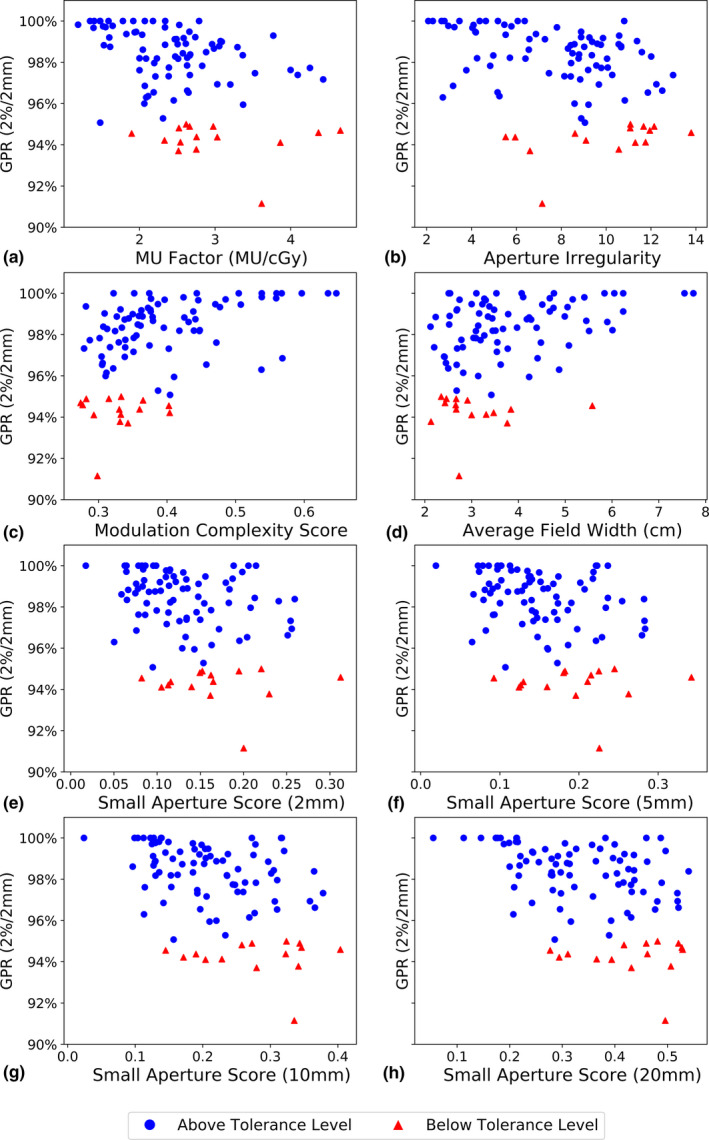
Complexity metrics evaluated for plans delivered on the TrueBeam linac plotted against gamma passing rate using 2%/2 mm. Plans with gamma passing rates above the 95% tolerance limit are denoted as blue circles, and plans with gamma passing rates below the tolerance limit are denoted as red triangles.

**Fig. 2:**
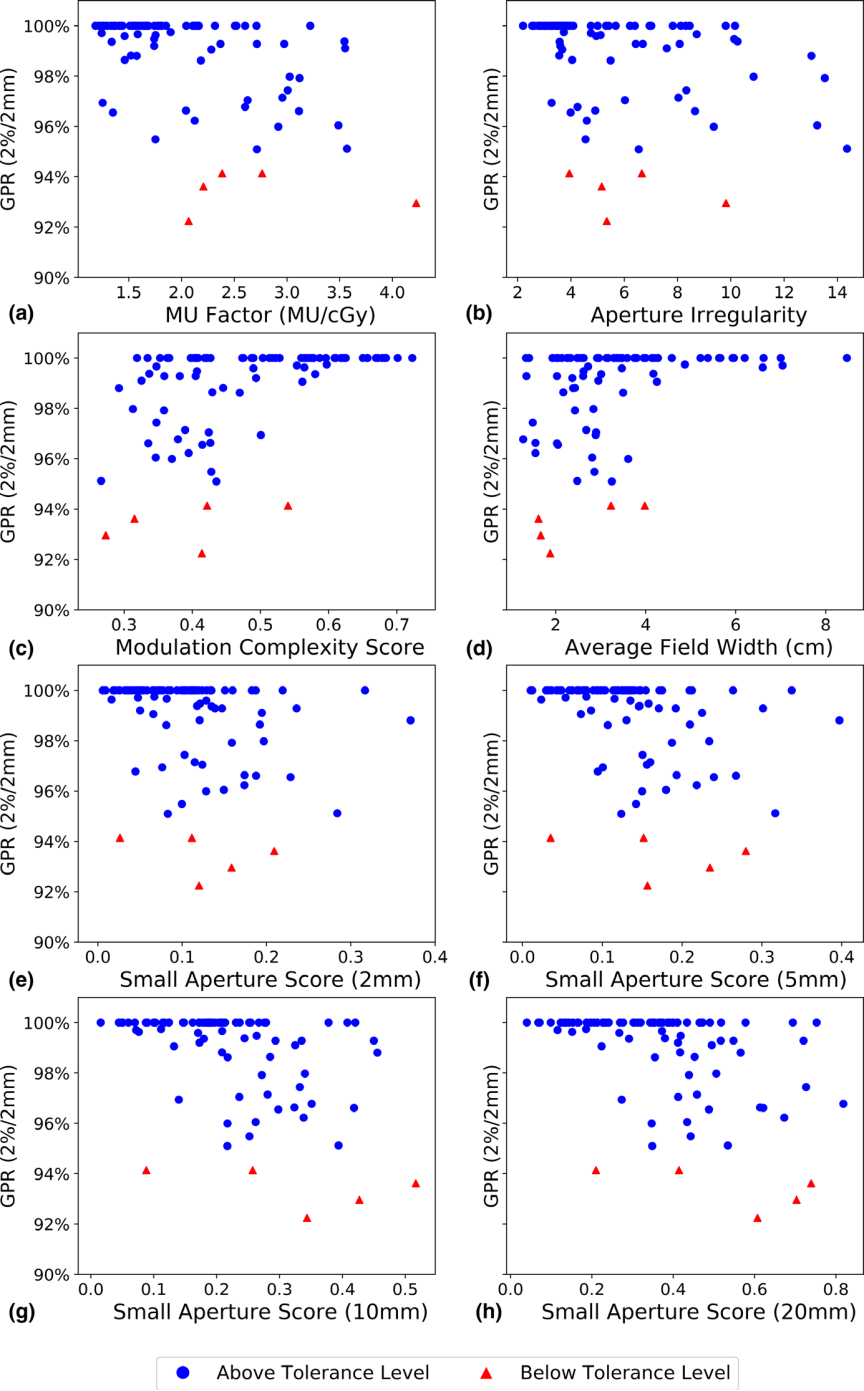
Complexity metrics evaluated for plans delivered on the TrueBeam STx linacs plotted against gamma passing rate using 2%/2 mm. Plans with gamma passing rates above the 95% tolerance limit are denoted as blue circles, and plans with gamma passing rates below the tolerance limit are denoted as red triangles.

Scatter plots of the complexity metrics and quality assurance results are presented for the TrueBeam and TrueBeam STx machines in Figs. 1 and 2, respectively. For similar levels of plan complexity, large variations in GPR were observed. In addition, the corresponding values of the correlation coefficient Spearman’s rho with significance are summarized in Table 4. Complexity metrics generally showed weak correlations to quality assurance results, and correlations found on the TrueBeam STx linacs were typically stronger than on the TrueBeam linac. For both types of linacs used, the MCSv was found to have moderate correlations to the GPR.

**Table 4:** Correlations of complexity metrics to gamma passing rate (2%/2 mm).

Complexity metric	TrueBeam r_s_ ^a^ (*P* value)	TrueBeam STx r_s_ ^a^ (*P* value)
MU factor (MU/cGy)	−0.444 (*P* < 0.001)	−0.472 (*P* < 0.001)
AI^b^	−0.455 (*P* < 0.001)	−0.489 (*P* < 0.001)
MCSv	0.502 (*P* < 0.001)	0.528 (*P* < 0.001)
Average field width (cm)	0.383 (*P* < 0.001)	0.318 (*P* = 0.002)
SAS^c^, 2 mm	−0.331 (*P* = 0.001)	−0.392 (*P* < 0.001)
SAS^c^, 5 mm	−0.345 (*P* < 0.001)	−0.470 (*P* < 0.001)
SAS^c^, 10 mm	−0.432 (*P* < 0.001)	−0.525 (*P* < 0.001)
SAS^c^, 20 mm	−0.478 (*P* < 0.001)	−0.477 (*P* < 0.001)

^a^ Spearman’s rho.

^b^ Aperture irregularity.

^c^ Small aperture score.

### 3.C Receiver operating characteristic curves

**Fig. 3:**
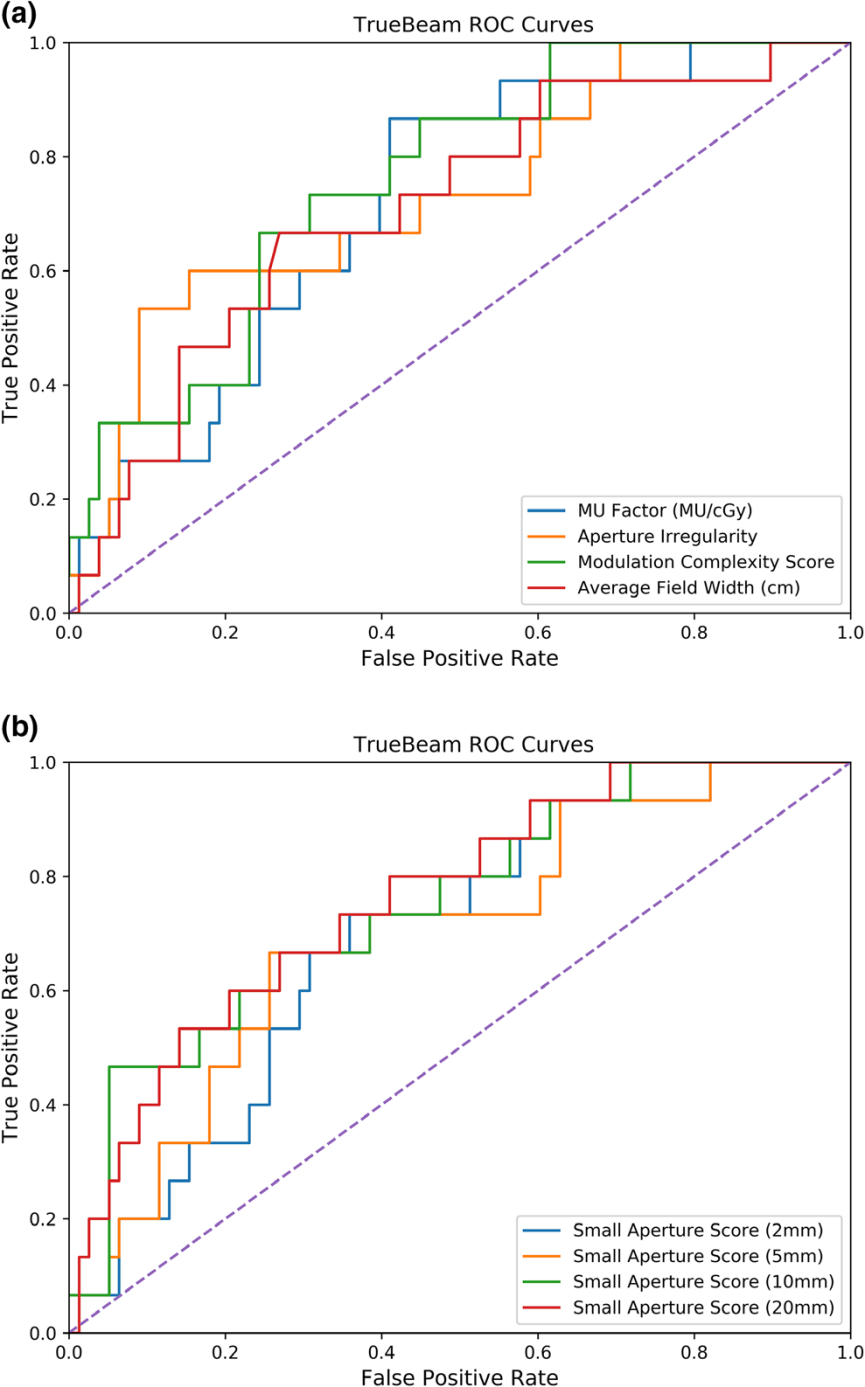
ROC curves for complexity metrics evaluated for plans delivered on the TrueBeam linac. The diagonal line represents random classification performance. (a) depicts the ROC curves for the MU Factor, Aperture Irregularity, Modulation Complexity Score, and Average Field Width, and (b) depicts the ROC curves for the Small Aperture Score, when the definition of small apertures is a leaf pair gap of 2, 5, 10, and 20 mm.

**Fig. 4:**
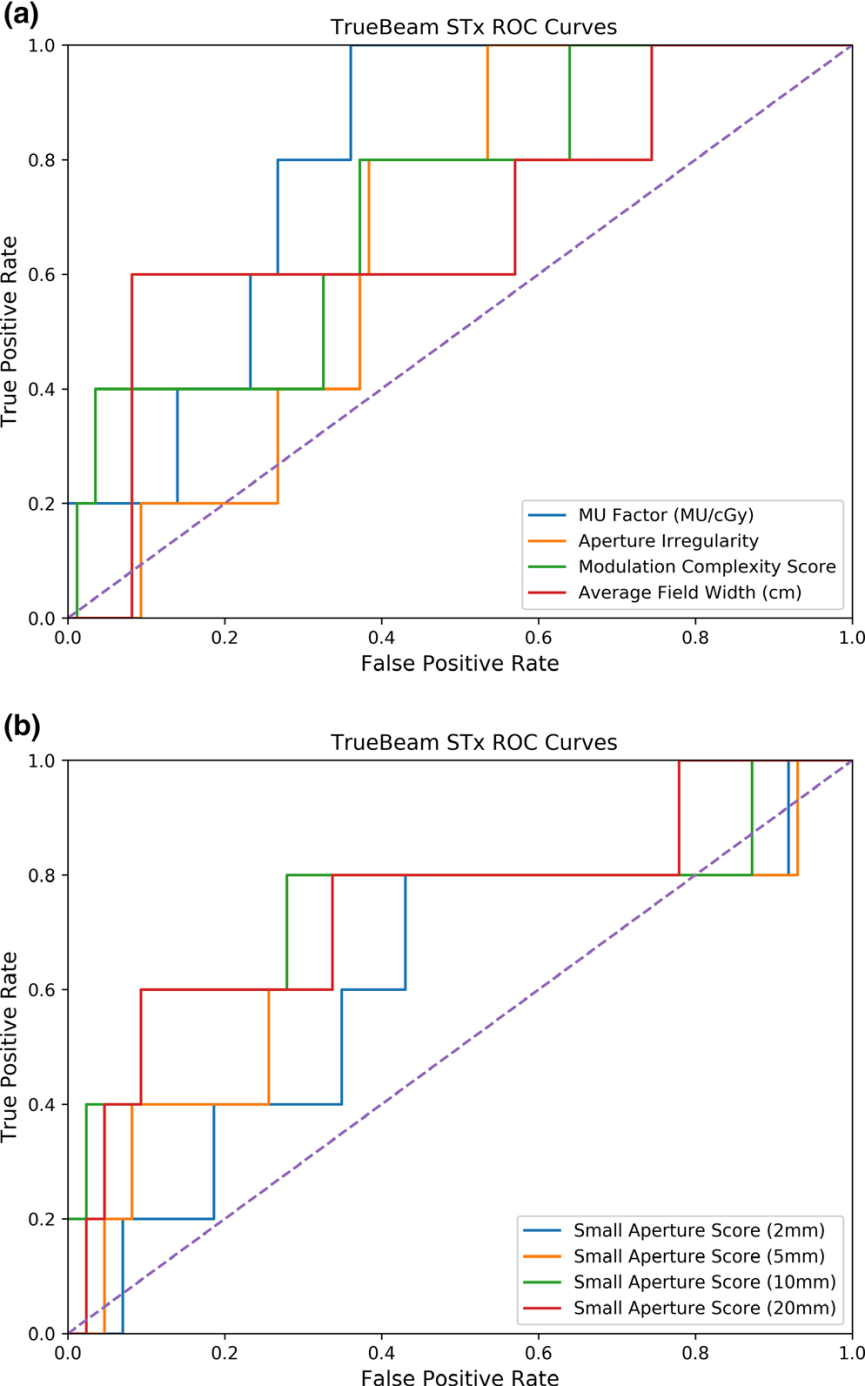
ROC curves for complexity metrics evaluated for plans delivered on the TrueBeam STx linacs. The diagonal line represents random classification performance. (a) depicts the ROC curves for the MU Factor, Aperture Irregularity, Modulation Complexity Score, and Average Field Width, and (b) depicts the ROC curves for the Small Aperture Score, when the definition of small apertures is a leaf pair gap of 2, 5, 10, and 20 mm.

Figures 3 and 4 depict the receiver operating characteristic curves for the TrueBeam and TrueBeam STx machines, respectively. Using the TrueBeam, the AI achieved the highest true positive rate of 53% with a corresponding false‐positive rate of 9%. Conversely, the average field width achieved a 60% true‐positive rate with an 8% false‐positive rate on the TrueBeam STx machines. All thresholds, true‐positive rates, and false‐positive rates can be found in Tables 5 and 6 for the TrueBeam and TrueBeam STx, respectively.

**Table 5:** TrueBeam threshold values and classification performance.

Complexity metric	Threshold	True positive rate	False positive rate
MU factor (MU/cGy)	3.62	27%	6%
AI^a^	11.07	53%	9%
MCSv	0.30	33%	4%
Average field width (cm)	2.46	27%	8%
SAS^b^, 2 mm	0.22	20%	6%
SAS^b^, 5 mm	0.24	20%	6%
SAS^b^, 10 mm	0.32	47%	5%
SAS^b^, 20 mm	0.48	40%	9%

^a^ Aperture irregularity.

^b^ Small aperture score.

**Table 6:** TrueBeam STx threshold values and classification performance.

Complexity metric	Threshold	True positive rate	False positive rate
MU factor (MU/cGy)	4.23	20%	0%
AI^a^	9.82	20%	9%
MCSv	0.32	40%	3%
Average field width (cm)	1.88	60%	8%
SAS^b^, 2 mm	0.21	20%	7%
SAS^b^, 5 mm	0.24	40%	8%
SAS^b^, 10 mm	0.34	60%	9%
SAS^b^, 20 mm	0.61	60%	9%

^a^ Aperture irregularity.

^b^ Small aperture score.

Table 7 summarizes the areas under the curve for each complexity metric. The complexity metrics performed fairly similarly in discriminating between passing and failing plans. The MCSv and the SAS defined at 20 mm had the largest areas of 0.76, showing equal and fair performance on the TrueBeam. The areas calculated for the TrueBeam STx machines had larger standard errors, though complexity metrics also performed similarly. Of note, the MU Factor had a good performance with an area of 0.80.

**Table 7:** Areas under curve for receiver operating characteristic curve.

Complexity metric	TrueBeam AUC^a^	TrueBeam STx AUC^a^
MU factor (MU/cGy)	0.72	0.80
AI^b^	0.73	0.67
MCSv	0.76	0.72
Average field width (cm)	0.71	0.69
SAS^c^, 2 mm	0.69	0.61
SAS^c^, 5 mm	0.70	0.68
SAS^c^, 10 mm	0.75	0.75
SAS^c^, 20 mm	0.76	0.74

^a^ Area under curve.

^b^ Aperture irregularity.

^c^ Small aperture score.

## 4 DISCUSSION

This investigation found that when considering the 2%/2 mm gamma criterion, 84% of plans delivered on the TrueBeam yielded GPRs above 95%, and 95% of plans delivered on TrueBeam STx machines yielded GPRs above 95%. The 15 plans yielding GPRs below 95% on the TrueBeam were generally distributed across all treatment sites. In comparison, the 5 plans yielding GPRs below 95% on the TrueBeam STx comprised of small treatment sites. The high proportion of plans passing QA contrasts greatly with past works, as Masi et al.^6^ reported only 64% of plans yielded GPRs above 90% using the 2%/2 mm criterion, and Li et al.^8^ reported only 40% for the same criterion. The increased proportion of passing plans found in this study may be due to variations between institutions such as beam models, machines and detectors. Furthermore, it is not clear if dose interpolation on the evaluated distribution, which could improve gamma calculation accuracy, was performed in other studies.

The complexity metrics of plans delivered on each type of linac differ slightly, shown in Table 3. The TrueBeam STx linacs are often used for treatments with small fields or simply shaped targets, and greatly benefit from the higher resolution MLC used. These plans include simple prostate, brain, and lung SBRT plans. These cases are reflected in the smaller Average Field Width and higher SAS scores in comparison to the TrueBeam linac, which is used for a larger variety of treatments. However, distinctions in treatment site were not considered for analysis as previous in‐house analysis found that GPRs could vary greatly within a disease site. For instance, while intact prostate plans tended to yield GPRs close to 100%, prostate plans with nodes tended to have lower GPRs. In addition, this study used two beam‐matched TrueBeam STx linacs, and variations between these linacs are an additional source of error that was not investigated.

Previous reviews of complexity metric correlations generally report weak to moderate correlations to quality assurance results,^3^, ^10^ as is the case in this work. The complexity metrics selected for this study were primarily used to describe the general MLC movement and aperture shape of treatments. The existence of correlations with the Pinnacle3 TPS and Varian linacs used at our institution suggests that extreme values of complexity metrics may indicate highly complex plans, and correspond to larger disagreements between the measured and calculated dose distributions. This trend coincides with results found in previous works, despite variations between institutions. Li et al.^8^ reported a moderate correlation of the Average Field Width to the GPR. Similarly, Masi et al.^6^ found a moderate correlation of the MCSv to the GPR. In addition, the AI yielded a moderate correlation to the dose difference between measurement and TPS calculations as reported by Du et al.^5^ However, correlation analysis was not always consistent. Masi et al.^6^ and Du et al.^6^ used Pearson’s correlation as opposed to Spearman’s correlation as used in this work, and individual institutions may use different measures of dose agreement.

As seen in Figs. 1(c) and 1(d), the MCSv and Average Field Width both had small ranges of values that overlap with the corresponding complexity metrics of failing plans. All failing plans had MCSv values <0.4, while the majority of failing plans also had Average Field Widths <4 cm. Given the relatively few plans yielding GPRs below the tolerance limit, these observations were not seen using the TrueBeam STx linacs. However, in both Figs. 1 and 2, complexity metrics at extreme values that suggest more complex treatments show a larger uncertainty in the quality assurance results. As such, based on the complexity metric used, extremely high or low values can suggest a larger disagreement between the measured and calculated dose distributions.

ROC curve analysis was also performed to investigate the classification performance of each complexity metric. The AUC is often used to represent the classification performance as a single value, ranging from 0.5 to 1 to indicate random classification and perfect classification, respectively. In this investigation, complexity metrics investigated in this work generally yielded AUCs between 0.7 and 0.8. In comparison, Park et al.^11^ reported the MCSv yielded an AUC of 0.527 using a 2%/2 mm criteria with a 90% tolerance limit, whereas the modulation index presented yielded an average AUC of approximately 0.8.

For the purpose of using complexity metrics as substitutes for dose verification measurements, a threshold value can be used to determine if the complexity of a treatment plan would indicate a high dose uncertainty. As a result, the given treatment plan may be considered for re‐planning. In this case, the threshold value should correspond to a low false positive rate to avoid flagging clinically acceptable plans and a high true positive rate to identify highly complex plans. However, any threshold value selected will be a compromise between the false positive rate and the true positive rate. The threshold values presented in Tables 5 and 6 were selected to ensure false positive rates did not exceed 10%. Younge et al.^12^ used the same constraint on the false positive rate and found that the author’s aperture complexity metric yielded a 44% true‐positive rate with a 7% false‐positive rate. In this work, the AI yielded a 53% true‐positive rate with a 9% false positive rate for the TrueBeam linac, whereas the average field width yielded a true positive rate of 60% and a false positive rate of 8% for the TrueBeam STx linacs.

The results of analyzing complexity metrics are highly institute dependent, thus making direct comparisons between institutions difficult. Quality assurance results are affected by the characteristics of the detector and phantom used for measurement, as well as the linac used for delivery, and the TPS used to generate treatment plans, particularly the accuracy of beam modeling. Analysis is further affected by the correlation method used, the criteria used for PSQA, as well as the number of treatment plans investigated and the corresponding treatment volume.^3^, ^10^ While specific results may not be applied to other institutions, the methodology can be used to develop institute specific PTQA tools to aid in the treatment planning process.

The values presented in Tables 5 and 6 are limited in their use to identify VMAT plans that may require re‐planning. All plans investigated in this study had been deemed clinically acceptable for delivery by the PTQA and PSQA at the institution. The failing plans presented are the result of using more stringent criteria for quality assurance as opposed to excessive modulation. Furthermore, due to the limited number of failing plans found on the TrueBeam STx linacs, the true positive rates reported in Table 5 may be more likely to be a product of chance than those reported in Table 4 for the TrueBeam linac. A larger sample size with a higher proportion of failing plans may result in a better indication of classification performance.^15^ Using the 3%/2 mm criterion, all plans investigated yielded GPRs above the tolerance limit. As a result, the 2%/2 mm criterion was required to show a larger range of GPRs.

In addition, the gamma analysis was performed by comparing two 2D dose distributions. While not performed for this study, a more accurate gamma analysis should compare a 2D measured dose distribution to a 3D plan dose distribution. The added dimension involved in gamma index calculations should result in better agreement between the dose distributions and higher GPRs in comparison to 2D gamma analysis.^16^ Future works should also include plans that are considered not suitable for clinical use, as well as plans with artificially high amounts of modulation created, to verify the classification performance of complexity metrics in clinical practice.

For studies of this nature, ROC analysis should be the preferred method of analysis. ROC analysis describes the classification performance of complexity metrics as PTQA tools, and can be used to assign threshold values for individual machines, target sites, or treatment techniques. In contrast, methods yielding single values such as correlation tests or the AUC do not fully represent the performance of complexity metrics. However, correlation tests and AUC analysis can still be insightful, and their results should coincide with one another. A lack of correlation or AUC values near 0.5 indicate that a given complexity metric cannot distinguish between treatment plans yielding higher or lower GPRs, and should not be considered for use. In general, the results found in this study coincided, suggesting that the complexity metrics investigated all have moderate capabilities to identify the degree of agreement between dose distributions.

## 5 CONCLUSION

This works investigated the potential use of complexity metrics as PTQA tools to compliment measurement‐based quality assurance at our institution. Complexity metrics can identify highly modulated plans that may require re‐planning without the need for dose verification measurements. Furthermore, complexity metrics can be used as a means of plan evaluation prior to physics check.

Most complexity metrics had weak correlations to PSQA results, with the exception of the MCSv which had a moderate correlation for both types of linacs considered. Using ROC analysis to investigate classification performance, the AI and the average field width were both found to have high true positive rates in identifying highly modulated plans, with corresponding false positive rates below 10%. The capacity for these complexity metrics to identify complex plans should be tested in future investigations. Treatment plans with artificial constraints on modulation, as well as those considered clinically unacceptable should also be incorporated in validation studies.

## CONFLICT OF INTEREST

No conflict of interest.

## References

[1] Miften M , Olch A , Mihailidis D , et al. Tolerance limits and methodologies for IMRT measurement‐based verification QA: recommendations of AAPM Task Group No. 218. Med Phys. 2018;45:e53–e83. 29443390 10.1002/mp.12810

[2] Otto K . Volmetric modulated arc therapy: IMRT in a single gantry arc. Med Phys. 2008;35:310–317. 18293586 10.1118/1.2818738

[3] Chiavassa S , Bessieres I , Edouard M , Mathot M , Moignier A . Complexity metrics for IMRT and VMAT plans: a review of current literature and applications. Br J Radiol. 2019;92:1–13. 10.1259/bjr.20190270 PMC6774599 31295002

[4] Crowe SB , Kairn T , Kenny J , et al. Treatment plan complexity metrics for predicting IMRT pre‐treatment quality assurance results. Australas Phys Eng Sci Med. 2014;37:475–482. 24810792 10.1007/s13246-014-0274-9

[5] Du W , Cho SH , Zhang X , Hoffman KE , Kudchadker RJ . Quantification of beam complexity in intensity‐modulated radiation therapy treatment plans. Med Phys. 2014;41:021716. 24506607 10.1118/1.4861821

[6] Masi L , Doro R , Favuzza V , Cipressi S , Livi L . Impact of plan parameters on the dosimetric accuracy of volumetric modulated arc therapy. Med Phys. 2013;40:071718. 23822422 10.1118/1.4810969

[7] McNiven AL , Sharpe MB , Purdie TG . A new metric for assessing IMRT modulation complexity and plan deliverability. Med Phys. 2010;37:505–515. 20229859 10.1118/1.3276775

[8] Li G , Wu K , Peng G , Zhang Y , Bai S . A retrospective analysis for patient‐specific quality assurance of volumnetric modulated arc therapy plans. Med Dosim. 2014;39:309–313. 24958705 10.1016/j.meddos.2014.05.003

[9] Nauta M , Villarreal‐Barajas JE , Tambasco M . Fractal analysis for assessing the level of modulating of IMRT fields. Med Phys. 2011;38:5385–5393. 21992358 10.1118/1.3633912

[10] Antoine M , Ralite F , Soustiel C , et al. Use of metrics to quantify IMRT and VMAT treatment plan complexity: a systematic review and perspectives. Phys Med. 2019;64:98–108. 31515041 10.1016/j.ejmp.2019.05.024

[11] Park JM , Park SY , Kim H , Kim JH , Carlson J , Ye SJ . Modulation indices for volumetric modulated arc therapy. Phys Med Biol. 2014;59:7315–7340. 25383976 10.1088/0031-9155/59/23/7315

[12] Younge KC , Roberts D , Janes LA , Anderson C , Moran JM , Matuszak MM . Predicting deliverability of volumetric‐modulated arc therapy (VMAT) plans using aperture complexity analysis. J Appl Clin Med Phys. 2016;17:124–131. 10.1120/jacmp.v17i4.6241 PMC5345484 27455504

[13] Valdes G , Scheuermann R , Hung CY , Olszanski A , Bellerive M , Solberg TD . A mathematical framework for virtual IMRT QA using machine learning. Med Phys. 2016;43:4323–4334. 27370147 10.1118/1.4953835

[14] Cancer Care Ontario . *Best Practice Guidance for Patient‐Specific Quality Assurance for IMRT and VMAT Plan Delivery Verification* . Ontario Health (Cancer Care Ontario). 2019 https://www.cancercareontario.ca/en/guidelines‐advice/types‐of‐cancer/67271. Accessed February 20, 2020.

[15] Bujang MA , Adnan TH . Requirements for minimum sample size for sensitivity and specificity analysis. J Clin Diagn Res. 2016;10:YE01‐YE06. 10.7860/JCDR/2016/18129.8744 PMC5121784 27891446

[16] Pulliam KB , Huang JY , Howell RM , et al. Comparison of 2D and 3D gamma analyses. Med Phys. 2014;41:021710. 24506601 10.1118/1.4860195 PMC3977814

